# Mitochondrial DAMPs as a critical driver in the development of acute graft versus host disease and emerging mitochondria targeted therapeutic strategies

**DOI:** 10.3389/fimmu.2025.1740433

**Published:** 2026-01-13

**Authors:** Vasantharaja Raguraman, Yogamaya Divakar Prabhu, Gopal Viswanathan Velmurugan, Gerhard C. Hildebrandt, Senthilnathan Palaniyandi

**Affiliations:** 1 Division of Hematology and Medical Oncology, Department of Medicine, Ellis Fischel Cancer Center, University of Missouri, Columbia, MO, United States; 2 Spinal Cord and Brain Injury Research Center, Department of Neuroscience, University of Kentucky, Lexington, KY, United States

**Keywords:** DAMP (Damage Associated Molecular Pattern), Graft versus host disease (GVHD), hematopoietic cell transplantation (HCT), mitochondrial DNA, mitochondria transfer

## Abstract

Mitochondrial fusion and fission regulate mitochondrial morphology and homeostasis, both of which are essential for maintaining cellular health. Free mitochondria and mitochondrial-containing extracellular vesicles have emerged as key mediators of pathological processes. Conditioning regimens for allogeneic hematopoietic cell transplantation (HCT) damage and lead to impaired mitochondrial function, including biogenesis and respiration, as well as elevated reactive oxygen species (ROS), which contribute to the development of inflammatory conditions as well as activation of antigen presenting cells, the latter being key players in acute graft versus host pathophysiology (GVHD). This leads to increased T-cell activation and proliferation, which increases alloreactivity and drives GVHD. Dysregulated mitochondrial dynamics lead to the release of mitochondrial DNA and formylated peptides, which act as Damage-Associated Molecular Patterns (DAMPs) and trigger cellular homeostatic imbalances, ultimately leading to more inflammation. The understanding that mitochondrial dysfunction contributes to GVHD offers novel therapeutic strategies, including blocking DAMP signaling and modulating immune cell metabolism to restore mitochondrial health. This review aims to understand mitochondrial homeostasis in both recipient and donor cells. This is crucial for understanding GVHD pathophysiology and developing mitochondria-targeted therapies or mitochondrial transfer strategies as potential therapeutic interventions for GVHD.

## Graphical Abstract

**Figure:**
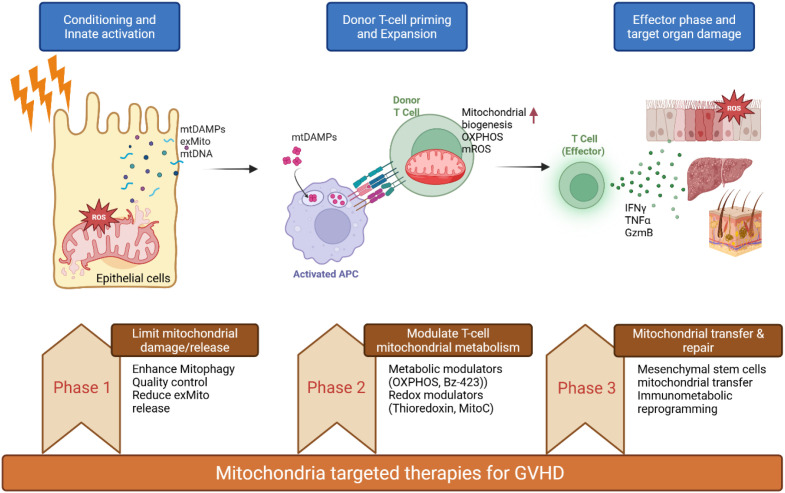

## Introduction

Allogeneic hematopoietic cell transplantation (HCT) offers a potential cure for various blood cancers and non-malignant blood conditions (1). Despite its benefits, the procedure is complicated by the development of acute graft-versus-host disease (GVHD) (1). Acute GVHD is a potentially life-threatening complication following allogeneic hematopoietic stem cell transplantation, often resulting in considerable morbidity and mortality. Acute GVHD develops through a complex set of immune interactions during allogeneic transplantation, occurring when donor-derived T lymphocytes recognize the recipient’s tissue as non-self due to disparities in histocompatibility, resulting in an immune-mediated attack. This typically happens within the first one hundred days after transplantation and can result in damage to multiple organs, such as the liver, gastrointestinal tract, skin, and lungs (2). Acute GVHD most frequently follows allogeneic bone marrow transplantation, but it can also happen after transplantation of solid organs with abundant lymphoid tissue, such as the liver, or after transfusion of nonirradiated blood. For the development of GVHD, immunocompetent cells must be transferred into a recipient with a weakened immune system, and these cells must recognize the host’s alloantigen (2).

Several factors influence the likelihood and severity of GVHD:

1. Donor and recipient factors: The risk of GVHD is higher with mismatched donors, differences in human leukocyte antigen (HLA) types, and sex mismatches.
2. Stem cell source and T cell content in the transplant product
3. Immune modulation and GVHD prophylaxis
4. Chemotherapy and radiation: Tissue injury caused by high-dose chemotherapy leads to elevated cytokine levels and cytokine storm that amplifies donor immune cell recognition of host antigens. Conditioning regimens that include total body irradiation further exacerbate the risk and severity of GVHD (3).

Despite major progress in the field, aGVHD remains the second most common cause of fatal illness in allo-HCT patients, surpassed only by disease relapse (4).

Acute GVHD occurs in about 30-60% of individuals who receive an allogeneic HCT, which primarily affects the gastrointestinal tract, skin, and liver (1). The development of acute GVHD is driven by antigen presentation from both blood-derived and other types of cells (5), chemokine activity in affected tissues, inflammatory responses triggered by damage-associated molecular patterns (DAMPs), and the recruitment of effector cells (6), and T cells to the sites of tissue injury (7). The risk of acute GVHD is linked to the intensity of the conditioning regimen, as more aggressive conditioning leads to the release of inflammatory signals from dying or damaged cells, which in turn activate the immune system and intensify acute GVHD (8).

Systemic corticosteroids are the standard treatment for grades II to IV acute GVHD, providing effective immunosuppression in about 35 to 60% of cases (1). However, those who fail to attain a lasting, thorough response to this first-line therapy are more likely to see their acute GVHD worsen, which raises their risk of death (9). Additionally, the severity of GVHD at the time of diagnosis has been linked to the overall survival rate (1).

## Mitochondria: beyond bioenergetics

Mitochondria serve as the powerhouse of the cell, producing adenosine 5’-triphosphate (ATP) through oxidative phosphorylation (OXPHOS) (10). Research over the past decade has revealed that mitochondria exist as a dynamic network within the cell, actively maintaining the quality of the mitochondrial population through fission, fusion, and selective degradation via mitophagy. Mitochondria also play crucial roles in regulating intracellular signaling pathways, producing reactive oxygen species (ROS), carrying out fatty acid β-oxidation, participating in amino acid metabolism, pyridine synthesis, phospholipid modification, calcium homeostasis, and influencing cell survival, aging, and death (11). To support these varied and vital cellular functions, mitochondria continuously undergo fission and fusion, which preserves their structure, network, and distribution within the cell. This ongoing renewal is crucial for maintaining mitochondrial health and overall cell function. When these processes become disrupted, it can lead to a broad spectrum of diseases, namely metabolic and neurodegenerative disorders, cardiovascular and inflammatory conditions, blood diseases, and cancer (10).

Maintaining a healthy cell requires precise control over mitochondrial quantity and functions to protect mitochondrial DNA (mtDNA) and ensure sufficient energy supply for cellular activities (11). Mitochondria are not formed anew in eukaryotic cells; instead, existing mitochondria are passed on to the two daughter cells during cell division (12). These organelles contain their mtDNA, and the process of mitochondrial biogenesis includes the replication, transcription, and translation of genes encoded by mtDNA, the movement of phospholipids between organelles, and the import of proteins produced in the nucleus into mitochondria via specialized translocation systems in mitochondrial membranes (13).

Mitochondrial homeostasis relies on the careful coordination of two opposing mechanisms: the creation of new mitochondria through mitochondrial biogenesis and the removal of damaged ones via mitophagy (11). Key molecules involved in this regulation include the peroxisome proliferator-activated receptor-γ coactivator (PGC-1α), which is the principal driver of mitochondrial biogenesis (11); the PTEN-induced putative kinase 1 (PINK1)-Parkin pathway (14), which plays a role in eliminating damaged mitochondria through a process known as mitophagy (15).

ATP, the cellular energy carrier, is generated by mitochondria in eukaryotic cells through OXPHOS, following the processing of the products generated from lipolysis and glycolysis. Furthermore, mitochondrial enzymes play a crucial role in various biosynthetic processes, including the production of steroids, nucleotides, lipids, hemes, and cholesterol, as well as maintaining ion homeostasis and regulating amino acid metabolism. Mitochondria are essential regulators of cell death pathways, and they also communicate via reactive oxygen species (ROS) and Ca2+. Due to their crucial role in signaling, metabolism, and bioenergetics, it is essential to tightly regulate the mitochondrial function and mass. Above all, the mass, activity, and shape of mitochondria vary widely between cell types and are dynamically regulated by a variety of physiological signals, including exposure to infectious agents, temperature, physical activity, circadian cues, and nutrient availability (15).

The mitochondrial membrane potential (ΔΨm) is generated through the redox reactions of the Krebs cycle and serves as an intermediate energy reservoir utilized by ATP synthase for ATP production. These processes establish both an electrical potential through charge separation and a proton gradient, which together contribute to the formation of the transmembrane hydrogen ion potential (16). Under stress conditions such as ischemia or inhibition of the electron transport chain, it can depend on ATP hydrolysis driven by the reverse rotation of the ATP synthase’s F1 subunit (17). Preserving the ΔΨm is essential for proper mitochondrial function, as it underlies protein import, ion balance, and numerous other biochemical processes within the organelle (18–20). The regulation of ΔΨm involves both the activity of proton pumps and the control of ΔΨm dissipation. This dissipation can either power ATP synthesis, which is linked to various ATP hydrolysis-dependent energy-consuming reactions (21), or result in heat generation through uncontrolled ion leakage across the inner mitochondrial membrane, without capturing the energy for productive use (22). Extensive research has documented the outcomes of Δψm dissipation, encompassing mitochondrial dysfunction resulting from impaired protein import, reduced ATP synthesis, or disrupted Ca2+ homeostasis, as well as its role as a trigger for mitophagy. In contrast, the implications of elevated ΔΨm remain comparatively understudied (23).

**Figure 1:**
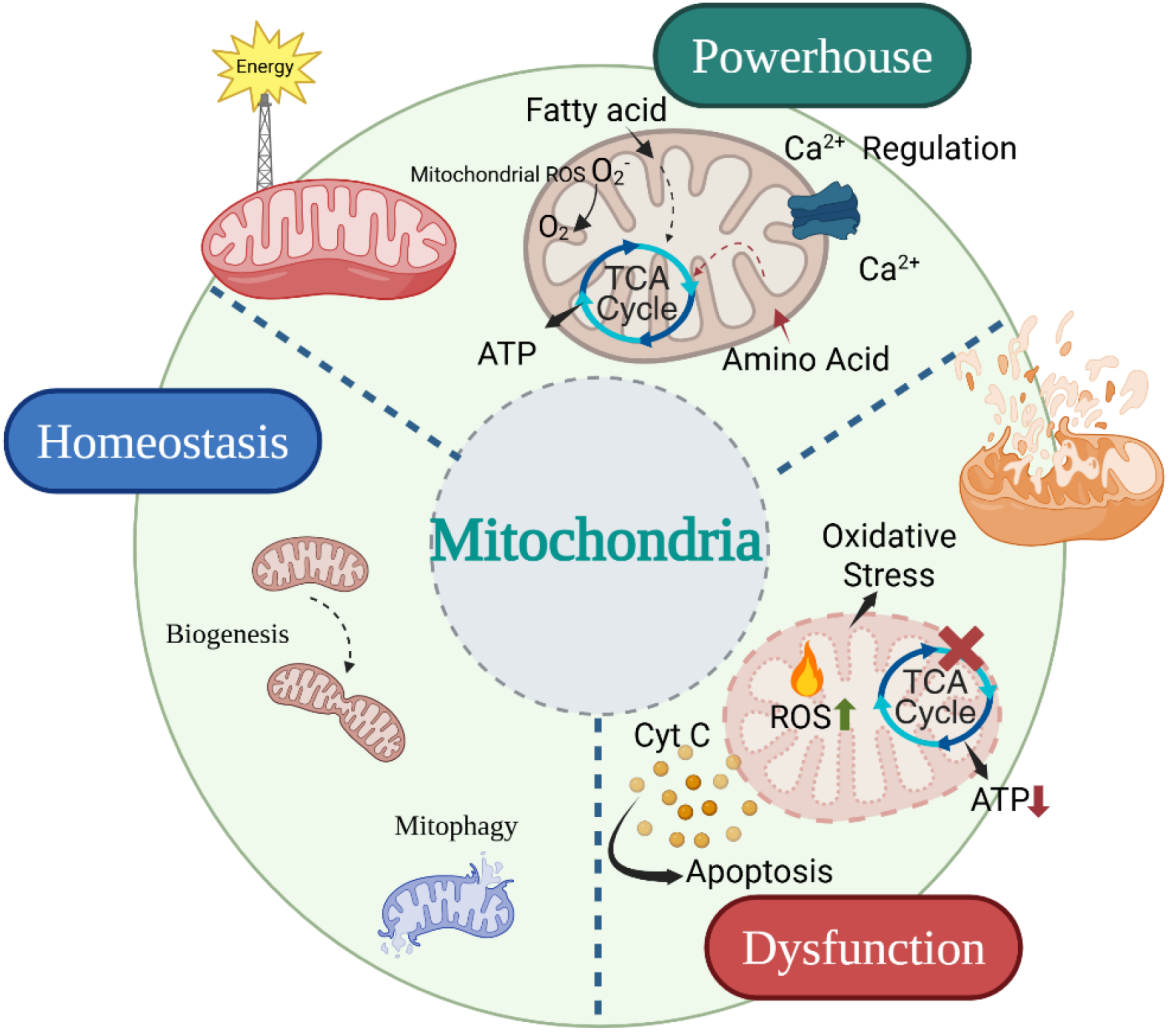
Role of altered mitochondrial bioenergetics and biogenesis leads to disease pathology. This figure was created using BioRender (biorender.com).

Mitochondria are two-membraned organelles that contain their own genetic material, which codes for a small but crucial portion of the mitochondrial proteome. The nuclear DNA encodes for most mitochondrial proteins, and various complementary mechanisms ensure the proper assembly, targeting, processing, and import of these proteins into the mitochondria. The organization of expression between the mitochondrial and nuclear genomes is crucial for the proper construction and function of the OXPHOS system, which is essential for energy metabolism in all eukaryotic cells. *In Vivo*, mitochondria exhibit high levels of mobility and connectivity. Mitochondrial dynamics, a vital physiological mechanism, ensure the proper positioning of mitochondria at intracellular locations with high energy consumption. Mitochondria are also essential for the biosynthesis of heme, iron-sulfur clusters, and other prosthetic groups. Additionally, mitochondria play a significant role in the production of nucleic acids, amino acids, and lipids. An essential location of ROS synthesis is the mitochondrial respiratory chain; several enzymes are required to strictly regulate the amount of ROS produced. Metabolic diseases are caused by an imbalance between the generation of ROS and detoxification (**Figure 1**). Therefore, mitochondrial malfunction has been linked to metabolic diseases, and mitochondrial activity is crucial for the cell’s fate (24).

## Mitochondrial biogenesis

Mitochondrial biogenesis refers to the process by which cells increase both the size and number of mitochondria in response to increased energy requirements and other signals (25). Since most mitochondrial proteins are encoded by nuclear DNA, precise coordination between the expression of nuclear and mitochondrial genes is necessary to successfully produce new mitochondria (26). Mitochondrial precursor proteins synthesized in the cytoplasm are transported into mitochondrial subcompartments through the TOM/TIM complex system. However, assembling these imported nuclear-encoded subunits with mitochondrial-encoded components can pose challenges (10). Accumulation of unassembled respiratory chain subunits correlates with heightened ROS production and mitochondrial proteotoxic stress, both of which negatively impact cellular function (10). To address these issues, cells employ multiple regulatory mechanisms, including specialized signaling pathways and transcription factor complexes (often assisted by coactivators) that coordinate mitochondrial biogenesis (27). Beyond transcriptional regulation, mitochondrial retrograde signaling molecules such as ATP, ROS, and calcium ions (Ca^2+^) also modulate this process (28). Notably, disturbances in mitochondrial membrane potential impair mitochondrial Ca^2+^ uptake, leading to elevated cytosolic Ca^2+^ levels. This calcium surge activates Ca2+-dependent kinases, triggering signaling cascades that regulate mitochondrial biogenesis (10).

The first nuclear transcription factors identified as regulators of mitochondrial biogenesis were nuclear respiratory factors 1 (NRF1) and 2 (NRF2) (10). NRF1 functions as a positive regulator of transcription due to its C-terminal activation domain, which enables it to bind to the promoters of mitochondrial-associated genes (29). Extensive research has established NRF1 as the principal regulator of mitochondrial biogenesis (11). NRF1 governs the expression of genes involved in respiratory chain complexes, enzymes for heme biosynthesis, elements required for mitochondrial DNA transcription and replication, as well as proteins essential for mitochondrial transport and assembly (30). Cytochrome C was the first mitochondrial gene shown to be upregulated by NRF1, with a specific NRF1 binding site identified upstream of its promoter, which is crucial for optimal promoter activity (29).

NRFs help mediate communication between nuclear and mitochondrial processes by upregulating constituents of the mitochondrial protein import system, including TOMM20, TOMM34, and TOMM701 (31). NRF1 activation also induces the expression of mitochondrial transcription factors TFAM, TFB1M, and TFB2M, which directly regulate mtDNA transcription, replication, and stability. These mechanisms ensure synchronized nuclear-mitochondrial coordination during mitochondrial biogenesis (10). NRF2 plays a crucial role in controlling the transcription of genes involved in the mitochondrial respiratory chain and mitochondrial gene expression. Genes encoding subunits of Cytochrome C Oxidase (COX) and other respiratory chain components have been found to contain functional NRF2 binding sites (30). While NRF2 can bind to the TFAM promoter, its effect on TFAM promoter activity is less pronounced than that of NRF1 (32). Typically, gene promoters contain binding sites for both NRF1 and NRF2, suggesting these factors work together to regulate mitochondrial biogenesis (30).

The major regulatory molecules involved in mitochondrial biogenesis, PGC-1α, NRF1/2, and TFAM, play critical roles in preventing the release of damage-associated molecular patterns (DAMPs). The release of mitochondrial constituents, such as mitochondrial DNA (mtDNA), is known to elicit robust pro-inflammatory responses. Moreover, the conditioning regimen administered prior to HSCT contributes to tissue injury and facilitates the onset of GVHD.

The interplay between tissue damage and GVHD involves the release of microbial and tissue derived factors – pathogen-associated molecular patterns (PAMPs) and DAMPs- that activate host antigen-presenting cells (APCs) (33). Also, activating the innate immune pathways, which play a central role in GVHD. Guided by this perspective, this review aims to provide a comprehensive overview of the emerging role of mitochondrial biology in the pathogenesis and regulation of GVHD.

## Mitochondrial DAMP signals associated with GVHD

Mitochondria play a crucial role in the production of ATP, which is essential for cellular energy supply; however, they are also involved in the activation and differentiation of immune cells in various disease conditions. The activation of immune cells requires substantial energy to perform their effector functions, such as producing cytokines or chemokines. For instance, immune cells such as activated T and B cells rely on glycolysis, whereas regulatory T cells or M2 macrophages largely depend on mitochondrial function to meet their energy demands (39). Mitochondria also regulate the immune response, in addition to being important sources of energy, as a defense against infections and cancer. The role of mitochondrial damage-associated molecular patterns (mtDAMPs), such as mtDNA, damaged mitochondria, and proteins, has been recognized as a critical factor in maintaining and regulating immune responses in multiple immunological conditions (39).

**Figure 2:**
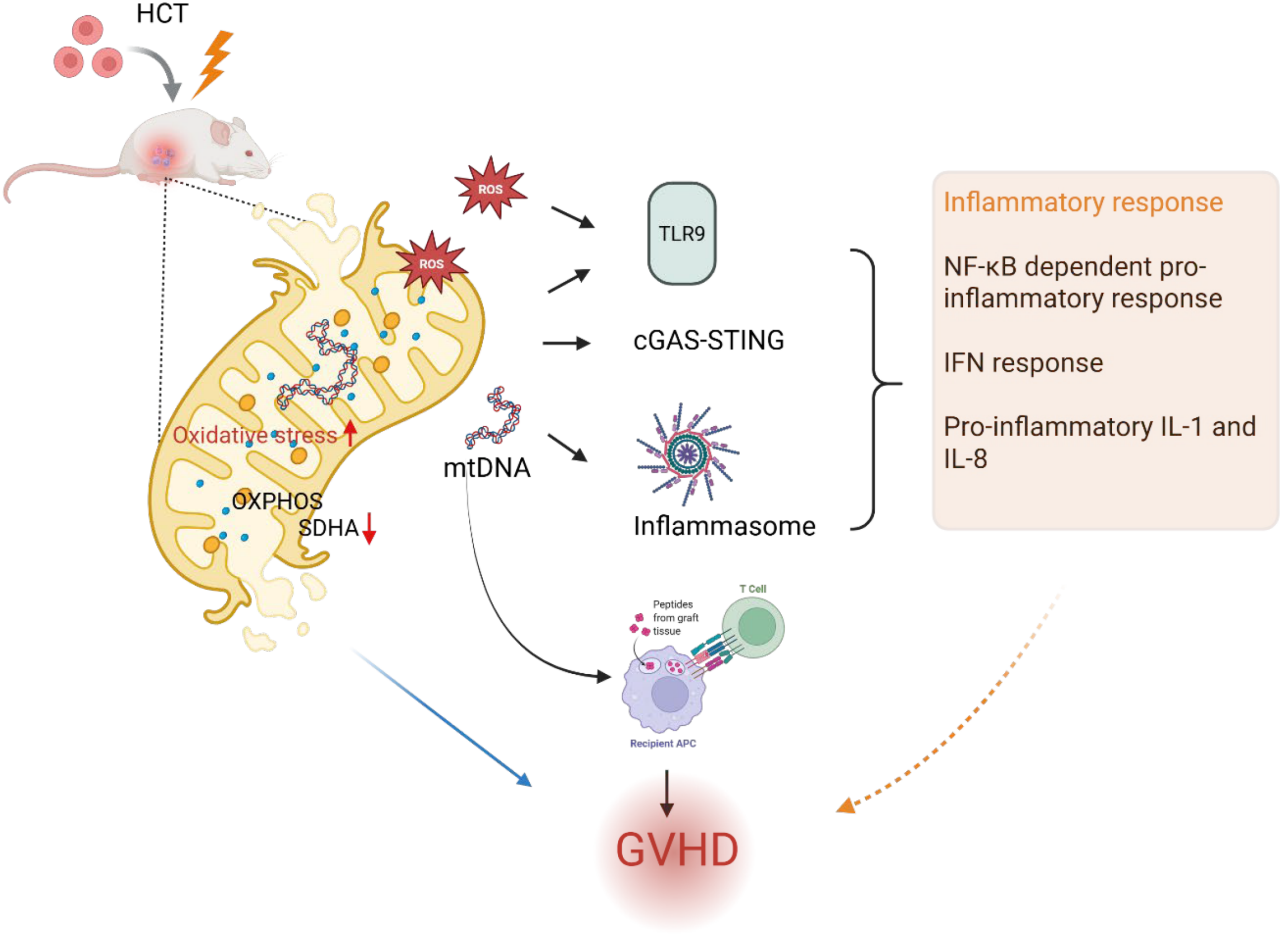
Mitochondrial dysfunction pathways contributing to Graft Versus Host Disease. This figure was created using BioRender (biorender.com).

Mitochondria possess distinct features that make them highly immunogenic organelles, enabling them to activate the immune system through pattern recognition receptors (PRRs). The release of mitochondrial products such as mtDNA, cardiolipin, and mitochondrial formylated peptides can act as DAMPs recognized by PRRs and trigger the innate immune response (40). Herein, we discuss how dysfunctional mitochondria or alterations in the structure can trigger inflammation in the context of GVHD (**Figure 2**, **Table 1**). Mitochondrial DNA (mtDNA) is present in every cell except erythrocytes, which lack mitochondria. mtDNA is highly susceptible to oxidative damage and exhibits low methylation levels, enveloped into protein-DNA complexes known as nucleoids. The release of mtDNA is sensed by innate immune PRRs, and their interactions initiate a pro-inflammatory signaling cascade (41). Multiple studies suggest that circulating mtDNA is elevated in multiple human diseases reviewed by Giordana et al. (42). Recently, several studies have explored how mtDNA escapes from the matrix into the cytosol, such as mitochondrial apoptosis mediated by mitochondrial outer membrane permeabilization (MOMP) initiates the rupture of the mitochondrial membrane and mtDNA release in the cytosol (43–45). The release of mitochondrial DNA and other mtDAMPs during early conditioning activates host APCs, thereby enhancing donor T-cell proliferation and differentiation into effector cells, which potentiate inflammatory responses mediating GVHD development. Cell-free mitochondrial DNA in plasma functions as an endogenous antagonist against TLR9, with elevated levels that augment the B-cell response, and may serve as a biomarker and mechanistic driver contributing to chronic GVHD (37). In contrast, decreased mtDNA copy numbers in T-lymphocytes have been associated with acute GVHD, presenting a potential biomarker to identify individuals with aGVHD. However, further studies are needed to accurately interpret T cell mtDNA and extracellular mtDNA release findings (46).

**Table 1:** Key preclinical findings on the role of mitochondrial metabolism in GVHD.

Study	Model	Key Mitochondrial Findings	Impact on GVHD pathophysiology	References
Pre-HSCT conditioning and extracellular mitochondria	Murine model (C57BL6/J into BALB/c) + pediatric cohort	Cytotoxic conditioning triggers the release of extracellular mitochondria, resulting in impaired complex I-dependent ATP generation and oxidative damage.	The activation of host antigen presenting cells by circulating extracellular mitochondria enhances alloreactive donor T-cell responses, contributing to the severity of GVHD.	(33)
Bioenergetics of alloreactive T-cells	Murine Model (B6-Thy1.1 into B6D2F1)	Alloreactive donor T cells post-HSCT showed upregulated oxidative phosphorylation (OXPHOS) alongside glycolysis, hyperpolarized mitochondrial membrane potential, and overall increased mitochondrial activity.	Altered mitochondrial metabolism supports the survival, proliferation, and effector function of alloreactive T cells mediating GVHD.	(34)
Alloreactive T-cells and mitochondrial reactive oxygen species (ROS)	Murine model (B6 into BALB/c and B6 into BD2F1)	Alloantigen-activated T cells show increased mitochondrial ROS generation.	Mitochondrial ROS serve as second messengers to amplify T-cell activation in GVHD, suggesting that enhancing antioxidant/redox regulatory pathways can attenuate T–cell–mediated organ damage.	(35)
Mitochondrial redox regulation by Sirt3 in donor T cells	Murine model (B6 into BALB/c and B6TCDBM+ *SIRT3^−/−^*T into BALB/c)	Sirt3, a mitochondrial deacetylase, regulates ROS and antioxidant defenses; loss of Sirt3 in donor T cells unexpectedly reduces GVHD severity.	Disruption of mitochondrial redox balance in donor T cells can lead to a detrimental GVHD response, highlighting sirt3-dependent mechanisms as attractive immunometabolic targets.	(36)
Circulating cell-free mitochondrial DNA (mtDNA) as DAMPs	Adult and pediatric cohorts	Children at risk for late aGVHD/cGVHD exhibit elevated plasma cf-mtDNA, which serves as an endogenous TLR9 ligand and associates with severe GVHD.	Circulating cell-free mtDNA acts as a mitochondrial DAMP that enhances B-cell and innate responses, coupling tissue damage to chronic inflammation and GVHD pathogenesis, and offering as a potential biomarker and therapeutic target.	(37)
Translational strategies targeting mitochondrial function				
Mitochondrial Redox Regulation	Murine model (B6 into BALB/c and B6 into BD2F1)	Redox regulators, such as thioredoxin-1, limit mitochondrial ROS accumulation and the proliferation of alloreactive T cells and IFN-γ production.	Targeting the ROS pathway is beneficial in reducing alloreactive T cell response in GVHD without impairing the graft-versus-leukemia effect.	(35)
Modulating mitochondrial bioenergetics	Murine Model (B6-Thy1.1 into B6D2F1)	Pharmacological inhibition of mitochondrial F_1_F_0_-ATPase using small molecule inhibitor Bz-423 affects mitochondrial respiration.	Mitochondrial ATPase/ETC inhibitors and modulating ROS and bioenergetics selectively induce apoptosis in alloreactive T-cells and ameliorate GVHD.	(34)
Mitochondrial transfer	Humanized xenogenic‐GVHD mouse model	Mesenchymal stem cell-mediated transfer of mitochondria (MitoT) increases the expression of mRNA transcripts involved in T-cell activation and T regulatory cell differentiation.	The transfer of MitoT reduced the degree of tissue inflammation and injury in the GVHD mouse model, suggesting that organelle-based therapies may be a promising approach.	(38)

A recent study showed that the extracellular release of damaged mitochondria (exMito) induced by the conditioning regimen exacerbates GVHD through host APC activation mediated by donor T-cell activation in aGVHD. Extracellular mitochondria induce upregulation of MHC-II, costimulatory molecules such as CD86, and pro-inflammatory cytokine production in host APCs, leading to augmented donor T-cell activation and exacerbating GVHD. Moreover, plasma analysis from conditioned mice and a pediatric HSCT cohort revealed conditioning induced extracellular release of mitochondria, suggesting the response is conserved across experimental and clinical settings (33).

## Mitochondrial ROS

Mitochondria, the metabolic center of the cell, serve to fulfill cellular demands by receiving signals that trigger their functions. Nevertheless, gathering evidence suggests that mitochondria can actively provide signals by releasing proteins, lipids, metabolites, and ROS as signaling molecules. The generation of reactive oxygen species (ROS) by mitochondria via the electron transport chain (ETC) can affect immune cell functions in multiple ways (47). For instance, ROS acts as a second messenger in multiple signaling pathways, such as the Ca^2+^ NFAT signaling pathway, which is decisive in T cell activation (48). Conditioning regimens with chemotherapy/radiation increase systemic ROS and loss of antioxidant defenses, generating an oxidizing environment that promotes activation of antigen-presenting cells and triggers the early phase of acute GVHD. Neutrophil-derived ROS exacerbate epithelial injury and barrier disruption in the gut, which correlates with poor outcomes, underscoring that early ROS amplify subsequent donor T cell mediated tissue damage (49). Upon allogenic stimulation, the sustained generation of mitochondrial ROS served as second messengers for T-cell activation, proliferation, and cytokine production (48, 50, 51). Alloantigen-activated T cells exhibit elevated mitochondrial ROS compared to non-alloreactive cells, linking the tight association between the mitochondrial ROS and effector T cell responses in acute GVHD (51). Therefore, maintaining redox balance and reducing oxidative stress appear to be essential in preventing GVHD. Sirtuin 3 (SIRT3), a mitochondrial histone deacetylase (HDAC), plays a crucial role in maintaining cellular processes by regulating reactive oxygen species (ROS) through enhanced antioxidant scavenging mechanisms. Interestingly, the loss of SIRT3 in alloreactive T cells reduced aGVHD by reducing ROS, likely a consequence of deficient T cell activation while preserving graft versus tumor effects (36). The administration of thioredoxin-1 (Trx1) counteracts oxidative stress by limiting ROS accumulation in donor T-cells through downstream signaling, including NF-κB activation, reduced proliferation, and decreased GVHD severity, suggesting that targeting the ROS pathway is beneficial in treating aGVHD (35).

## Mitochondrial energy metabolism defects

T cells in a resting state depend on mitochondrial respiratory capacity and OXHPOS for their metabolic and bioenergetic needs. When they are activated, their increased energy needs are largely satisfied through glycolytic pathways rather than mitochondrial OXPHOS. Activated T cells primarily rely on glycolysis, like cancer cells, to meet their energy demands and support the differentiation of effector T cells (52). In GVHD, alloreactive T cells undergoing differentiation into effector T cells with metabolic reprogramming depend on both glycolysis and induction of OXPHOS. It has been shown that proliferating T cells in response to alloantigens exhibited increased mitochondrial membrane potential (ΔΨm) and superoxide production, along with reduced levels of antioxidants (34). Conversely, another study reported that allogenic T cell activation highly depends on glycolytic activity, as indicated by the buildup of glycolytic intermediates, higher levels of LDH-A, Mct4, Glut1, and Glut3 mRNA, glucose-6-phosphate levels, and increased ECAR levels compared to syngeneic bone marrow transplantation (53). This suggests that T cells undergo distinct metabolic reprogramming in response to alloantigen *in vitro* and *in vivo* conditions and require further investigation for targeting energy metabolism. Furthermore, the T-cell-mediated damage in intestinal epithelial cells (IEC) is regulated by mitochondrial complex II during gastrointestinal GVHD. IECs exhibited a reduction in OXPHOS and accumulated succinate due to a decrease in succinate dehydrogenase A (SDHA), a crucial component of mitochondrial complex II, leading to an aggravated disease severity in gastrointestinal GVHD (54). In addition, fatty acids (FA) contribute to alloreactive T cell proliferation by fueling mitochondrial OXPHOS to achieve maximal mitochondrial respiration (55, 56). Blocking FA oxidation impaired the survival of alloreactive T cells, suggesting the importance of FA metabolism may represent a promising therapeutic target for treating GVHD (56).

## Mitochondrial dynamics in immune cells

Mitochondrial fusion and fission are dynamic (morphology and network processes of mitochondria) processes critical for maintaining mitochondrial homeostasis and cellular health. It plays a vital role in energy production, cell differentiation, and cell division. Physiological and pathological conditions that induce increased energy demand trigger mitochondrial fission to meet these energy requirements (57, 58). However, uncontrolled excessive mitochondrial fission can lead to pathological mitochondrial fragmentation associated with mitochondrial dysfunction and reactive oxygen species (ROS) production, impairing the organelle’s ability to meet cellular energy demands and contributing to disease progression (59). Mitochondrial dynamics are tightly linked to mitochondrial bioenergetics in both cell-mediated and antibody-mediated immune cells. Mitochondrial dynamics are strongly associated with the metabolic status of the cell (60). It is also true that any intervention that perturbs mitochondrial dynamics in turn affects the oxidative phosphorylation (OXPHOS) and metabolism (61). The mitochondrial morphology and bioenergetic profile of immune cells, including macrophages, T cells, and B cells, adapt dynamically according to their activation status (61–63). Intriguingly, activation of T cells and macrophages, key mediators of cell-mediated immunity, is associated with increased mitochondrial fragmentation and reduced oxidative phosphorylation OXPHOS (61, 62). In contrast, activation of B cells, which mediate humoral immunity, also induces mitochondrial fragmentation but is accompanied by enhanced OXPHOS activity (64). Recently, a study demonstrated that alloreactive donor T-cells exhibit a shift in mitochondrial dynamics toward excessive mitochondrial fission via the Sphk1-dependent S1P/S1PR1 signaling pathway in aGVHD models. The downstream signaling pathway enhances DRP1 activation and mitochondrial fragmentation in allogeneic CD4 T cells through the AMPK/AKT/mTOR/DRP1 axis, further promoting a shift toward highly fragmented mitochondria that support pathogenic expansion and effector functions. Pharmacological inhibition of Sphk1 or S1PR1 significantly reduced GVHD severity while retaining graft-versus-leukemia effects (65). Interestingly, enhancing mitochondrial fusion can mitigate GVHD by promoting the suppressive function of Tregs in murine GVHD and *in vitro* models. Inhibition of acetyl-CoA carboxylase-1 (ACC1) in Tregs enhances mitochondrial fusion through FAO-mediated augmentation of OXPHOS and improves GVHD outcomes, suggesting that Treg-mediated prevention of GVHD is a potential therapeutic approach (66). These observations highlight a close relationship between the metabolic and functional states of immune cells and their mitochondrial dynamics. Despite growing recognition of the central role of mitochondria in immune regulation, there are only limited studies addressing mitochondrial function in GVHD. Little is known about how mitochondrial morphology, dynamics, and metabolism are altered in GVHD across different organs and immune cell subsets. This gap highlights the need for appropriate animal models and advanced tools to track, isolate, and functionally characterize both host- and donor-derived mitochondria during GVHD pathogenesis.

## Tools to study mitochondrial function and dynamics, and transfer *in vivo*

Most high-resolution studies of mitochondrial function and dynamics have been performed *in vitro*, primarily because mitochondria can be easily labeled with dyes such as MitoTracker. However, studying mitochondrial dynamics *in vivo* remains significantly more challenging. One major limitation of dye-based approaches is the potential for MitoTracker to leak and nonspecifically stain adjacent cells, which can confound the interpretation of intercellular mitochondrial movement. Historically, electron microscopy has been the primary tool for visualizing mitochondrial morphology and dynamics *in vivo*.

Recent evidence highlighted the emerging role of free mitochondria and extracellular vesicles containing mitochondria (EV-mito) as key pathological mediators in GVHD (33, 67). Understanding the mitochondrial homeostasis of both host and donor cells is therefore critical for elucidating the pathophysiology of GVHD and for developing mitochondria-targeted therapeutic interventions. To address the limitations of traditional approaches, transgenic animal models expressing mitochondria-targeted fluorophores have been developed, providing powerful tools to track mitochondrial dynamics with high spatial and temporal resolution. These genetically encoded reporters enable cell- or organ-specific mitochondrial labeling, enabling *in vivo* imaging through two-photon microscopy or *ex vivo* imaging in organotypic slices. Furthermore, mitochondria from specific cell types can be isolated from these transgenic animals to assess cell-specific mitochondrial function. For instance, mice expressing mitochondria-targeted photoactivatable Dendra2 (mtD2) (68) or green fluorescent protein (mtGFP) (69) can be crossed with tissue-specific Cre lines to investigate mitochondrial dynamics, trafficking, and function in defined cell populations *in vivo* (70). Importantly, these models also offer unique opportunities to study emerging phenomena such as mitochondrial transfer and transplantation (71), concepts that have not yet been explored in the context of GVHD.

## Spontaneous mitochondrial transfer and therapeutic mitochondrial transplantation

Intercellular communication is essential for maintaining homeostasis in multicellular organisms and can occur through direct cell–cell interactions, such as intercellular junctions, or via extracellular vesicles, including exosomes and microvesicles (72). Approximately two decades ago, organelle transport was recognized as an additional mode of intercellular communication, mediated primarily through transient, filamentous membrane structures known as tunneling nanotubes (TNTs), which bridge the cytoplasm of adjacent cells (72). *In vitro* studies have shown that TNTs facilitate the transfer of multiple organelles, including the Golgi apparatus, endoplasmic reticulum, and mitochondria, between different cells. Currently, five pathways have been identified through which spontaneous mitochondrial transfer can occur: extracellular vesicles (EVs), TNTs, gap junction channels (GJCs), mitochondria extrusion, and cell fusion (73). *In vivo* evidence has further established the physiological relevance of mitochondrial transfer. In the lung, mesenchymal stem cells (MSCs) and bone marrow–derived stem cells (BMSCs) donate mitochondria to airway epithelial cells, conferring protection against injury, inflammation, and mitochondrial dysfunction (74, 75). In the central nervous system, Hayakawa and colleagues demonstrated astrocyte-to-neuron mitochondrial transfer following focal cerebral ischemia (76). Even under normal physiological conditions, astrocytes transfer mitochondria to brain endothelial cells and pericytes, with this process increasing during aging (71). Mitochondrial transfer also plays detrimental or adaptive roles in disease. In cancer, mitochondria-depleted tumor cells acquire mitochondria from the surrounding stromal environment to restore bioenergetics and regain tumorigenic potential (77). Hence, the spontaneous mitochondrial transfer process that happens at physiological conditions provides a theoretical basis for the therapeutic potential of mitochondrial transplantation.

According to the latest recommendation of mitochondrial transfer and transplantation nomenclature and characterization, mitochondrial transplantation (MT) is a procedure in which mitochondria are isolated from a cellular or tissue source and then administered to an animal with the intent of eliciting a therapeutic response (78). Injection of mitochondria from healthy rats to diabetic rats improved myocardial function and fibrosis (79). In addition, direct injection of mitochondria into the muscle, liver, and spleen has also been shown to improve functional recovery (80–82). Intravascular injection of mitochondria has also been shown to improve cardiac function; however, the efficiency is lower because mitochondria are distributed throughout the body (83). Court et al. (38) demonstrated that transfer of mitochondria from mesenchymal stem cells to T cells induces Treg differentiation and reduces inflammation. Mechanistically, the transfer of Mito T to CD4^+^ T cells leads to a significant increase in a highly suppressive CD25+ FoxP3+ population in the mouse model of GVHD, suggesting the potential for exploring organelle-based therapies in immune cells. Similarly, allogeneic bone marrow (BM)- derived mesenchymal stem cells (MSCs) co-cultured with activated CD8+ T cells reduced their expansion and IFN-γ production by transferring mitochondria to the CD8^+^ T cells. The transfer of mitochondria from MSC to CD8^+^ T cells resulted in downregulation of T-bet and Eomes expression, leading to suppression of CD8^+^ T cells (84). Earlier studies have demonstrated that in GVHD, circulating cell-free mtDNA is elevated due to mitochondrial damage in moderate to severe GVHD (37). Based on this study, we speculate that combining autologous MT with allogenic HCT therapy will be beneficial for mitigating T cell-induced inflammation. According to the NIH ClinicalTrials.gov, there are three completed clinical trials (NCT05330676, NCT03384420, NCT04976140) and three ongoing clinical trials (NCT06065852, NCT04998357, NCT07066267) on MT. Based on the completed clinical trials, MT demonstrates improvements; however, further investigation is required (73).

## Limitations of mitochondrial transplantation

MT, while promising, faces several essential challenges related to inconsistent mitochondrial persistence, unclear mechanisms of action, and the vulnerability of isolated mitochondria to extracellular environments. Ideally, exogenous mitochondria should integrate into endogenous mitochondrial networks and provide sustained bioenergetic support; however, experimental findings frequently contradict this expectation. Studies report only transient improvements in ATP production that do not persist over the long term following mitochondrial transplantation (85). Additionally, results from ischemic heart models show functional recovery within minutes after mitochondrial delivery, even though mitochondrial internalization and functional integration typically require several hours (86, 87). This discrepancy raises concerns that observed early improvements may stem from stress signaling or other indirect mechanisms rather than genuine mitochondrial uptake and activity. A further challenge is the uncertain stability of exogenous mitochondria in the extracellular space. Physiological concentrations of Ca²^+^ and Na^+^ in blood and interstitial fluid can rapidly damage isolated mitochondria, leading to structural disruption, loss of function, and release of mitochondrial components (88). Such components, including mtDNA and formylated peptides, can act as DAMPs, potentially provoking unwanted inflammation or systemic immune responses following intravascular delivery (89). Therefore, transplanted mitochondria may exert early protective or therapeutic effects through energy-independent mechanisms, such as activation of innate immune pathways or stress-response signaling. These findings collectively suggest that the therapeutic efficacy of MT may arise from complex downstream signaling cascades initiated shortly after transplantation rather than from long-term survival or integration of exogenous organelles. Consequently, it remains unclear whether beneficial outcomes depend on mitochondrial incorporation into host networks, DAMP-mediated immunomodulation, stimulation of endogenous mitochondrial biogenesis, or interactions among these mechanisms. Addressing these uncertainties is essential for advancing the field.

## Targeting mitochondrial metabolism

**Figure 3:**
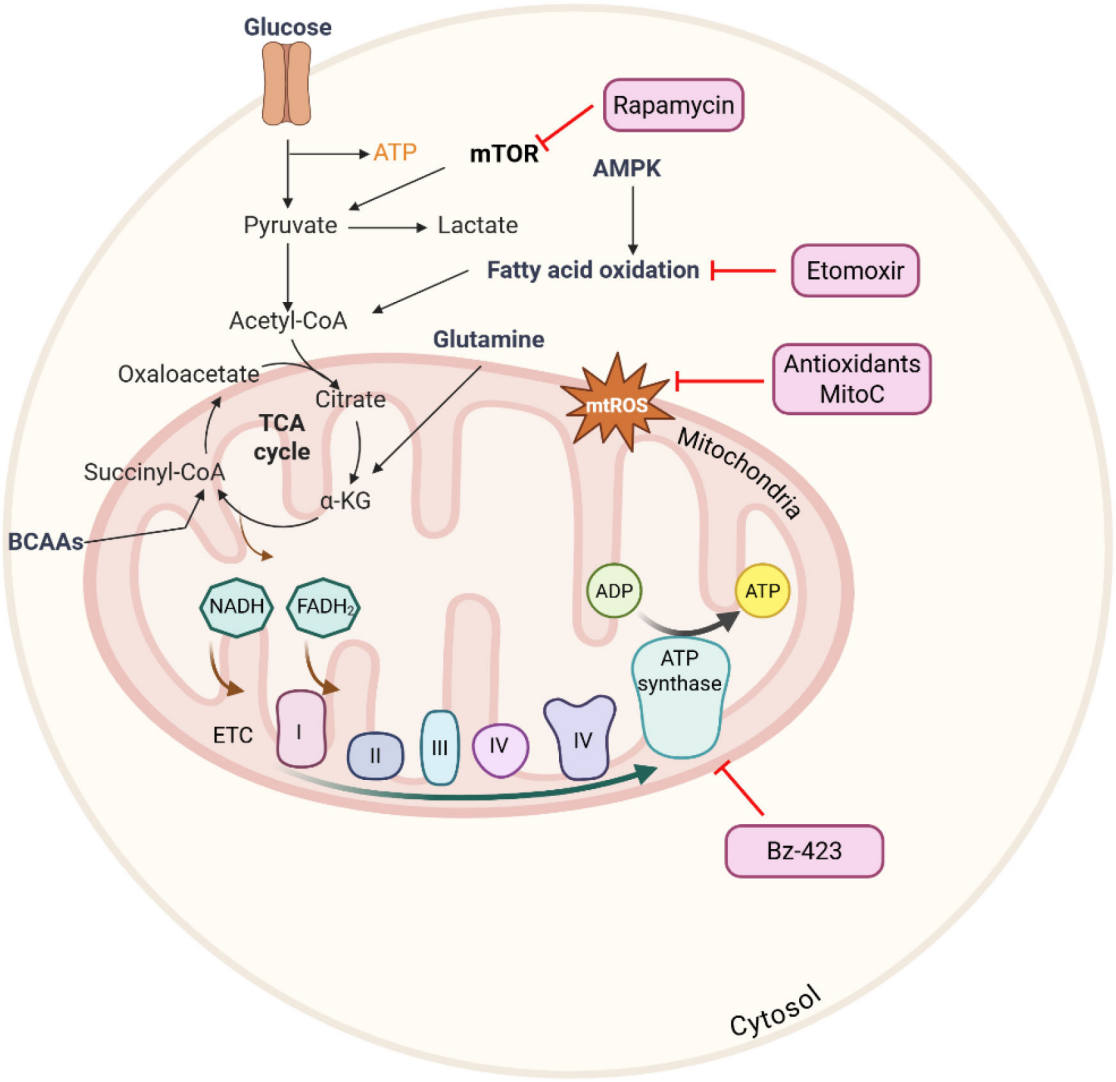
Mitochondrial metabolic pathways and their therapeutic targets in immune cells. This figure was created using BioRender (biorender.com).

The critical role of mitochondria in regulating the survival and death of immune cells makes them a promising therapeutic target in disease conditions. The strategies that target mitochondrial dysfunction include controlling mitochondrial ROS, oxidative stress levels, inhibition of ETC complexes, and OXPHOS or other proteins of mitochondrial membranes. The alloreactive T cells in GVHD highly rely on both aerobic glycolysis and OXPHOS, and the accumulation of acylcarnitines, along with changes in fatty acid oxidation. The distinct bioenergetic metabolism can be targeted by a small-molecule inhibitor, Bz-423, which targets mitochondrial F1F0 adenosine triphosphate synthase (F1F0-ATPase), resulting in enhanced superoxide and apoptosis induction in alloreactive T cells (34). The upregulation of fatty acid transport and FAO enzymes during GVHD indicates that alloreactive T cells may rely on fatty acids as a key metabolic fuel source. Treatment with etomoxir, a CPT1a inhibitor that blocks FAO, impaired the survival of alloreactive T cells without affecting the normal T cells reconstitution, indicating a metabolic dependency unique to alloreactive cells (56). The role of granzyme B (GzmB)-Serine protease inhibitor 6 (Spi6) axis is critical in allogeneic T cell response, due to the accumulation of Spi6 in mitochondria during allogeneic T cell activation. Loss of Spi6 in donor T cells disrupted mitochondrial homeostasis, as evidenced by altered mitochondrial membrane potential and elevated ROS levels, and prompted GzmB-dependent activation-induced cell death (AICD), predominantly via fratricide, which leads to reduced clonal expansion in the host and markedly ameliorated GVHD symptoms (90). These findings suggest that targeting mitochondrial bioenergetics and redox characteristics can be developed as a therapeutic strategy for mitigating GVHD (**Figure 3**).

## Mitochondria targeted antioxidants

Several studies have shown that developing nontargeted antioxidants using conventional methods for restoring physiological conditions during oxidative stress may not be successful in clinical trials, due to contradictory results and adverse effects. Now, the era is moving towards nontargeted antioxidants to target such mitochondrial targeted antioxidants, which might be beneficial in multiple disease conditions (91). The targeted delivery of antioxidants enables the protection of cells from oxidative stress by accumulating in cells and mitochondria. In recent years development of mitochondria-targeted antioxidants such as MitoE, MitoQ, MitoC, and MitoSOD has given promising results in protecting tissues from oxidative damage through multiple mechanisms (91). In an aGVHD model, the administration of mitochondrial-targeted curcumin (MitoC) resulted in reduced mitochondrial TrxR activity and glutathione levels, improved regulation of mitochondrial ROS (mROS) production, and limited the activation-induced increase in mitochondrial biomass. MitoC suppressed the alloreactive T-cell activation and induced Treg differentiation via Nrf2-dependent modulation and inhibited aGVHD (92).

## Conclusion and future directions

This review underscores the intricate relationship between mitochondrial function and immune regulation, and it has become clear that mitochondria serve as signaling platforms through DAMPs and ROS in GVHD. Mitochondria orchestrate the balance between metabolism and immune function to maintain immune metabolic homeostasis. However, many questions remain unanswered in the context of alloreactive responses and GVHD pathophysiology. These include a mechanistic understanding of the regulation of mitochondrial DAMPs, as well as their role in mediating GVHD, which requires further clarification. Several studies have reported the role of mitochondrial metabolism in alloreactive T cells in GVHD; however, whether mitochondria also affect other immune cells requires further investigation. Mitochondrial dysfunction can result in alterations in mitochondrial dynamics, biogenesis, morphology, oxidative stress, ROS production, metabolic activity, disruptions in mitophagy, and other damaging factors, ultimately accelerating the progression of disease. Modulation of mitochondrial metabolism and bioenergetics in donor alloreactive T cells is well characterized, yet substantial challenges remain in understanding how intrinsic differences in mitochondrial function between donor and recipient cells contribute to the development and severity of GVHD. Addressing these bidirectional mitochondrial interactions is critical, as therapeutic approaches that modulate both donor and host mitochondrial function may better maintain immune balance and mitigate GVHD pathology. In this context, we highlight the emerging role of mitochondria-derived DAMPs as key drivers of inflammatory amplification during GVHD progression, as well as the importance of *in vivo* tools that allow precise investigation of mitochondrial transfer, turnover, and dynamics between donor and recipient compartments. Furthermore, identifying mitochondrial transplantation strategies and mitochondria-targeted therapeutic interventions presents a promising avenue for developing innovative treatments that aim to restore metabolic homeostasis and reduce GVHD severity. Together, these perspectives highlight the importance of an integrated understanding of mitochondrial communication, stress signaling, and bioenergetic regulation in both donor and host cells, thereby advancing the development of mitochondrial-targeted next-generation GVHD therapies.
